# Very low-density lipoprotein receptor increases in a liver-specific manner due to protein deficiency but does not affect fatty liver in mice

**DOI:** 10.1038/s41598-021-87568-2

**Published:** 2021-04-13

**Authors:** Yui Oshio, Yuta Hattori, Hatsuho Kamata, Yori Ozaki-Masuzawa, Arisa Seki, Yasutaka Tsuruta, Asako Takenaka

**Affiliations:** 1 Department of Agricultural Chemistry, School of Agriculture, Meiji University, Kawasaki, Kanagawa 214-8571 Japan; 2 Department of Chemistry and Life Science, College of Bioresource Sciences, Nihon University, Kameino, Fujisawa, Kanagawa Japan

**Keywords:** Biochemistry, Physiology

## Abstract

Very low-density lipoprotein receptor (VLDLR) is a member of the LDL receptor family that is involved in the uptake of VLDL into cells. Increased hepatic VLDLR under endoplasmic reticulum (ER) stress has been shown to cause fatty liver. In this study, the effect of dietary protein restriction on hepatic VLDLR and the role of VLDLR in fatty liver were investigated using *Vldlr* knockout (KO) mice. Growing wild-type (WT) and KO mice were fed a control diet containing 20% ​​protein or a low protein diet containing 3% protein for 11 days. In WT mice, the amount of hepatic *Vldlr* mRNA and VLDLR protein increased by approximately 8- and 7-fold, respectively, due to protein restriction. *Vldlr* mRNA and protein levels increased in both type 1 and type 2 VLDLR. However, neither *Vldlr* mRNA nor protein levels were significantly increased in heart, muscle, and adipose tissue, demonstrating that VLDLR increase due to protein restriction occurred in a liver-specific manner. Increased liver triglyceride levels during protein restriction occurred in KO mice to the same extent as in WT mice, indicating that increased VLDLR during protein restriction was not the main cause of fatty liver, which was different from the case of ER stress.

## Introduction

Very low-density lipoprotein receptor (VLDLR) is a member of the low-density lipoprotein receptor (LDLR) family and is involved in lipid transport via apolipoprotein recognition^1^. In *Vldlr*-deficient mice, blood triglyceride (TG) concentration is high and lipid uptake into adipose tissue and adipose tissue mass are reduced, demonstrating that VLDLR has a function in the uptake of TG-rich VLDL into peripheral tissues^2–4^. Retinal angiomatous proliferation is also one of the major phenotypes in *Vldlr*-deficient mice^5^. Receptors of the LDLR family are composed of five domains: a ligand-binding domain, an epidermal growth factor (EGF) precursor homology domain, an O-linked sugar chain domain, a transmembrane domain, and a cytoplasmic domain^1^. While LDLR mainly recognizes apolipoprotein (Apo) B-100, VLDLR specifically recognizes ApoE in chylomicrons, VLDL, and intermediate-density lipoprotein that are rich in TG. VLDLR has also been reported to promote lipid uptake by increasing TG hydrolysis by lipoprotein lipase (LPL) and receptor-mediated endocytosis^2^.

VLDLR is highly expressed in tissues with active lipid metabolism, such as the heart, skeletal muscle, and adipose tissue^6^. VLDLR expression increases in response to various stimuli and induces lipid accumulation in these tissues. VLDLR expression induced by peroxisome proliferator-activated receptor (PPAR) γ promotes lipid accumulation in adipose tissue^7^. Increased VLDLR promotes lipid accumulation in ischemic heart in mice^8^ and *Vldlr* deficiency suppresses lipid uptake and insulin resistance in the muscle of leptin-deficient mice^9^.

The expression of VLDLR in the liver has been demonstrated to be lower than that in other organs^10,11^, and its function in the liver is not well understood. However, in recent years, studies on VLDLR expression in the liver and its role in lipid metabolism have progressed. Activation of activating transcription factor 4 (ATF4) during endoplasmic reticulum (ER) stress induces *Vldlr* gene expression, as well as fatty liver, and both of these inductions disappear in *Vldlr*-deficient mice^12^. Since liver lipid accumulation during ER stress also disappears in mice lacking ApoE, it is strongly suggested that VLDLR-mediated lipid uptake into the liver may be a major cause of hepatic fat accumulation during ER stress^12^. Alcoholic fatty liver has also been suppressed in *Vldlr*-deficient mice^13^, and fibroblast growth factor 21 (FGF21)-mediated increases in VLDLR cause hepatic steatosis^14^. In addition, fenofibrate administration causes an increase in liver VLDLR and a decrease in blood TG due to PPARα activation^15^. Thus, increased VLDLR expression in the liver likely causes an increase in liver fat.

Recently, the action of macrophage VLDLR in inflammation has also been studied. In adipose tissue, increased VLDLR expression in macrophages promotes adipose tissue inflammation and impaired glucose tolerance in obese mice^16^. Furthermore, adipose tissue inflammation in obese mice fed a high-fat diet is reduced in *Vldlr*-deficient mice^17^. In addition, VLDLR and soluble VLDLR extracellular domain (sVLDLR-N) released into blood guide leukocytes into tissues via binding to fibrin and cause an inflammatory reaction^18^. The reports that VLDLR induces inflammatory responses are accumulating; however, there are many points on the mechanism that require clarification.

*Vldlr* messenger RNA (mRNA) has two types of splicing variants, type 1 and type 2. These structural differences indicate that type 2-VLDLR lacks the O-linked glycosylation domain encoded by exon 16^10,19^. The two variant mRNAs also have different tissue distributions. Type 1 is expressed more in heart, muscle, and adipose tissue than type 2, while type 2 is expressed in non-muscle tissues, such as the cerebrum, kidney, spleen, adrenal gland, testis, ovary, and uterus^10,11^. The mechanism by which *Vldlr* mRNA splicing differs among tissues has not been clarified. It has also been reported that type 1 has a higher affinity for VLDL than type 2^10^. In addition, since the O-linked glycosylation domain enhances the stability of VLDLR on the cell membrane, type 2 lacking this domain is easily cleaved by protease and released outside the cell as sVLDLR-N^20^, which has been reported to affect intracellular Wnt signaling^11,21^.

A previous study conducted a DNA microarray analysis of protein-deficient rat liver and found that *Vldlr* gene expression is increased^22^. It has long been known that protein deficiency causes fatty liver, as well as growth retardation, and manifests in humans as a malnutrition symptom called kwashiorkor^23^. Therefore, in this study, the effect of protein deficiency on VLDLR and the role of VLDLR in fatty liver during protein deficiency in *Vldlr* KO mice were analyzed.

## Results

### Animal characteristics

**Figure 1:**
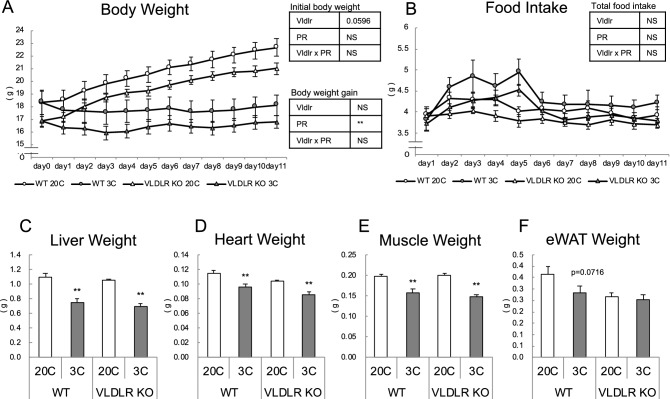
Effect of very low-density lipoprotein receptor (*Vldlr*) knockout and dietary protein restriction on growth in mice. (**A**) Body weight, (**B**) food intake, (**C**) liver weight, (**D**) heart weight, (**E**) gastrocnemius muscle weight and (**F**) epididymal white adipose tissue (eWAT) weight in wild type (WT) or *Vldlr*-deficient (VLDLR KO) mice fed control (20C) or protein-restricted (3C) diet. Values are the mean ± SEM, n = 10 per group. Results of two-way analysis of variance to evaluate the significant effects of *Vldlr* deficiency (*Vldlr*), protein restriction (PR), and the interaction of *Vldlr* × PR are shown in the tables; **P < 0.01, NS: not significant (**A**,**B**). Results of the difference between the two groups are shown. **P < 0.01, *P < 0.05 versus 20C (**C**–**F**).

The initial body weight of KO mice tended to be lower than that of WT mice, as reported previously (Fig. 1A). Body weight gain was lower in protein-restricted mice, while *Vldlr* deficiency did not affect body weight changes (Fig. 1A). Food intake was not influenced by either *Vldlr* deficiency or protein restriction (Fig. 1B). Liver, heart, and muscle weights were significantly (*P* < 0.01) lower in protein-restricted mice (Fig. 1C–E). The weight of epididymal white adipose tissue (eWAT) tended to decrease with protein restriction in WT mice, but did not change in KO mice (Fig. 1F).

### *Vldlr* mRNA in tissues

**Figure 2:**
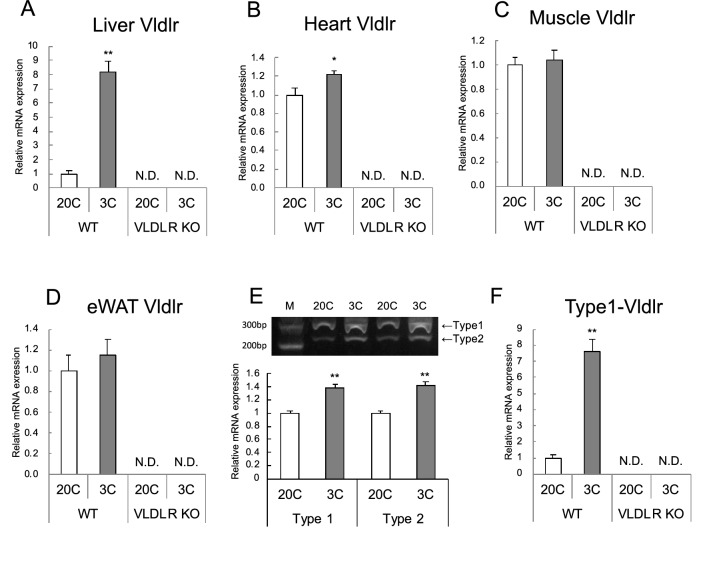
Effect of very low-density lipoprotein receptor (*Vldlr*) knockout and dietary protein restriction on *Vldlr* mRNA in mice. Relative *Vldlr* mRNA levels in (**A**) liver (total *Vldlr*), (**B**) heart, (**C**) gastrocnemius muscle, (**D**) epididymal white adipose tissue (eWAT), (**E**) liver (type 1 and 2) and (**F**) liver (type 1 *Vldlr*) in wild-type (WT) or *Vldlr*-deficient (VLDLR KO) mice fed a control (20C) or protein-restricted (3C) diet. M in (**E**) indicates a molecular marker. The unmodified full-length photograph of the gel in (**E**) is shown in Fig. S5. Values are expressed as a relative value with the WT 20C group as 1 and the mean ± SEM, n = 10 per group. **P < 0.01, *P < 0.05 versus 20C.

*Vldlr* mRNA levels were below detectable levels in all examined organs in KO mice. *Vldlr* mRNA increased in the liver and heart due to protein restriction in WT mice, and the increase was 1.2-fold higher in the heart, whereas the increase was approximately eightfold higher in the liver. *Vldlr* mRNA levels in muscle and eWAT were not affected by protein restriction (Fig. 2A–D).

### Type 1 and type 2 *Vldlr* mRNA in the liver

Reverse transcriptase-polymerase chain reaction (RT-PCR) analysis of the amount of type 1 and type 2 mRNA showed that both types increased significantly in the protein restriction group (Fig. 2E). In addition, when the relative amount of type 1 mRNA was quantitatively analyzed using real-time PCR, it was significantly increased with protein restriction (Fig. 2F). The amount of type 1 mRNA was measured using real-time PCR with exon 16-specific primers, but the amount of type 2 mRNA could not be measured because type 2-specific primers for real-time PCR could not be designed.

### Tissue VLDLR levels

**Figure 3:**
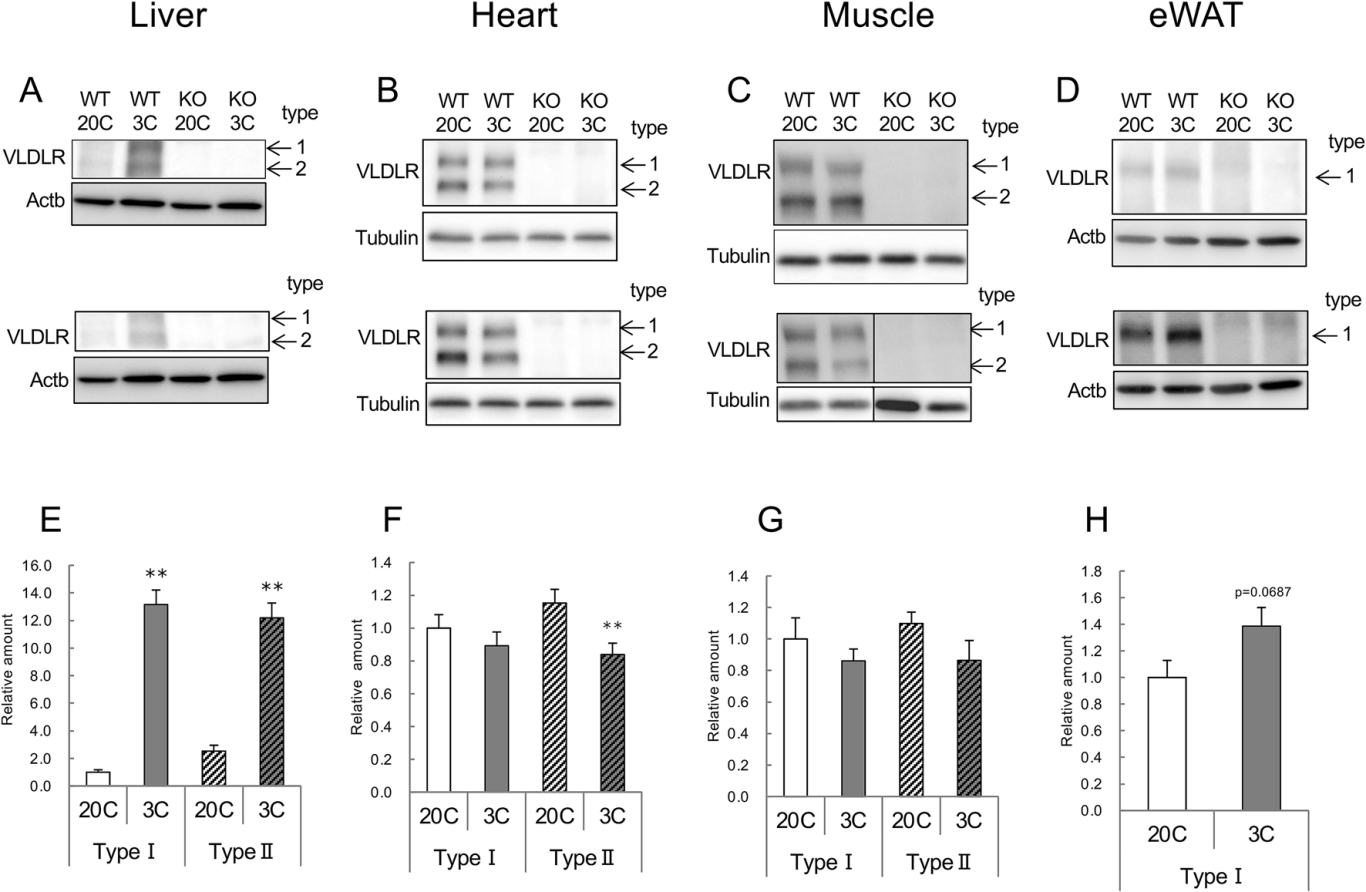
Effect of very low-density lipoprotein receptor (*Vldlr*) knockout and dietary protein restriction on VLDLR in mice. Typical western blotting images of VLDLR and loading control (Actb and Tubulin) in the liver, heart, gastrocnemius muscle, and epididymal white adipose tissue (eWAT) (**A**,**B**,**C**,**D**, respectively), and the total amount of type 1 and type 2 VLDLR (**E**,**F**,**G**,**H**) obtained by digitizing the band density of western blotting in wild-type (WT) or *Vldlr*-deficient (KO) mice fed the control (20C) or protein-restricted (3C) diet. Photographs of two samples are shown, and a black line in the lower image of muscle VLDLR and tubulin delineate the boundary between the gels processed in parallel. The brightness and contrast of the images depicting liver VLDLR, eWAT VLDLR and muscle tubulin were adjusted equally across the entire image. Unmodified full-length photographs of the blots are shown in Figs. S1 and S6. Values are expressed as a relative value with the WT 20C group as 1 and the mean ± SEM, n = 10 per group. **P < 0.01, *P < 0.05 versus 20C.

The amount of VLDLR was below the detection limit in *Vldlr*-KO mice (Fig. 3A–D). Type 1 and type 2 VLDLRs were detected in the liver, heart, and muscle, but only type 1 was detected in eWAT (Fig. 3A–D). Both type 1 and type 2 VLDLR levels were significantly increased in the liver due to protein restriction, but not in other organs (Fig. 3E–H). The amount of VLDLR in the liver was considerably lower than that in the heart, muscle, and eWAT in WT mice (Fig. S1).

### Asparagine synthase (Asns) mRNA in tissues

**Figure 4:**
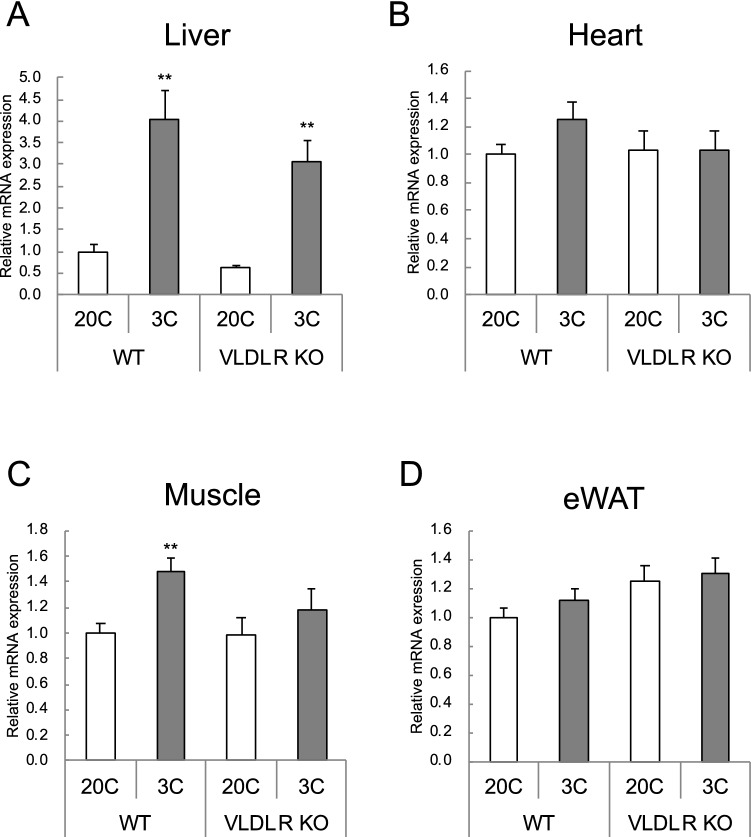
Effect of very low-density lipoprotein receptor (*Vldlr*) knockout and dietary protein restriction on *Asns* mRNA in mice. Relative *Asns* mRNA levels in (**A**) liver, (**B**) heart, (**C**) gastrocnemius muscle, and (**D**) epididymal white adipose tissue (eWAT) in wild-type (WT) or *Vldlr*-deficient (VLDLR KO) mice fed the control (20C) or protein-restricted (3C) diet. Values are expressed as a relative value with the WT 20C group as 1 and the mean ± SEM, n = 10 per group. **P < 0.01, *P < 0.05 versus 20C.

*Asns* mRNA levels were measured as a gene whose expression is induced by ATF4. *Asns* gene expression increased 3–4 fold in the livers of protein-restricted mice (Fig. 4A) and also increased in WT muscle due to protein restriction, but the rate of increase was only 1.5-fold (Fig. 4C). No increase in *Asns* mRNA was observed in heart and eWAT (Fig. 4B,D).

### Liver and plasma lipids

**Figure 5:**
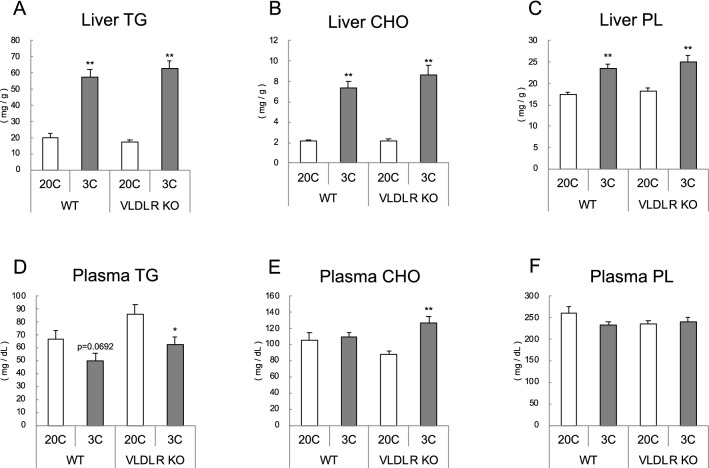
Effect of very low-density lipoprotein receptor (*Vldlr*) knockout and dietary protein restriction on liver and plasma lipids in mice. Liver and plasma triglyceride (TG) (**A**,**D**), cholesterol (CHO) (**B**,**E**), and phospholipid (PL) (**C**,**F**) in wild-type (WT) or *Vldlr*-deficient (VLDLR KO) mice fed the control (20C) or protein-restricted (3C) diet. Values are expressed as the mean ± SEM, n = 10 per group. **P < 0.01, *P < 0.05 versus 20C.

Hepatic TG, cholesterol, and phospholipid levels were increased by protein restriction, but were not affected by *Vldlr* deficiency (Fig. 5A–C). For plasma lipids, TG concentration decreased and cholesterol concentration increased with protein restriction only in *Vldlr*-deficient mice (Fig. 5D,E). The results of the two-way ANOVA analysis revealed that plasma TG levels were increased in VLDLR KO mice (effect of diet, *P* < 0.01; effect of VLDLR KO, *P* < 0.05; no interaction between the two factors). Plasma phospholipid concentration was not influenced by either protein restriction or *Vldlr* deficiency (Fig. 5F).

### Markers of liver injury and inflammation

**Figure 6:**
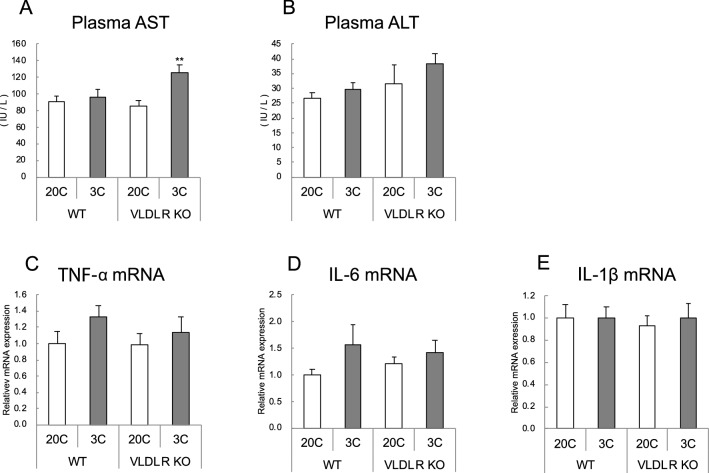
Effect of very low-density lipoprotein receptor (*Vldlr*) knockout and dietary protein restriction on plasma markers of hepatic injury and hepatic inflammatory cytokine mRNAs in mice. Plasma aspartate aminotransferase (AST) (**A**), alanine aminotransferase (ALT) (**B**), and liver mRNA levels of transforming growth factor-α (*TNF-α*) (**C**), interleukin-6 (*IL-6*) (**D**), and interleukin 1β (*IL-1B*) (**E**) in wild-type (WT) or *Vldlr*-deficient (VLDLR KO) mice fed the control (20C) or protein-restricted (3C) diet. mRNA level are expressed as a relative value with the WT 20C group as 1. Values are the mean ± SEM, n = 10 per group. **P < 0.01, *P < 0.05 versus 20C.

Plasma aspartate aminotransferase (AST) concentration increased with protein restriction only in *Vldlr*-deficient mice, while plasma alanine aminotransferase (ALT) concentration was not affected (Fig. 6A,B). Hepatic mRNA levels of inflammatory cytokines were not affected by either protein restriction or *Vldlr* deficiency (Fig. 6C–E).

### Effect of Fgf21 deficiency on *Vldlr* mRNA in the liver

The levels of hepatic *Vldlr* mRNA expression were increased by protein restriction in *Fgf21* KO mice to the same extent as in WT mice (Fig. S2).

### Effect of *ApoE* deficiency on liver fat

The increase in hepatic fat due to protein restriction was not suppressed in *ApoE*-deficient mice (Fig. S3).

### Changes in *Vldlr* mRNA content over time during protein deficiency

The increase in *Vldlr* mRNA expression began to appear on day 3 of protein deficiency, which was later than the onset of increased liver fat (Fig. S4).

## Discussion

In this study, *Vldlr*-deficient mice were used as experimental animals to analyze the role of *Vldlr* in protein restriction. The results of the two-way ANOVA analysis revealed that plasma TG levels were increased in VLDLR KO mice, which was consistent with previous reports^2–4^. According to the supplier, the Jackson laboratory, *Vldlr*-deficient mice show a slight decrease in body weight, body mass index, and epididymal fat mass^4^. In the KO mice used in this study, lower body weight was also observed, demonstrating that these mice had a phenotype consistent with the report. This low body weight is associated with a decrease in adipose tissue mass^4^. *Vldlr* deficiency did not affect growth retardation by protein restriction. Growth retardation due to protein restriction is caused by a decrease in body protein anabolism and is associated with a decrease in plasma levels of the anabolic hormone insulin-like growth factor-I^24,25^. The present results might reflect the hypothesis that growth retardation due to protein restriction and reduced weight due to *Vldlr* deficiency occurred by different mechanisms.

In the present study, it was revealed for the first time that the intake of a low-protein diet significantly increased hepatic *Vldlr* mRNA levels. Furthermore, the amount of *Vldlr* type 1 and type 2 mRNA was similarly increased by protein restriction. Regarding the regulation of type 1 and type 2, there is little information other than the tissue distribution of the two types of receptors^10,11^. It has also been reported that type 1 has a higher binding affinity to the ligand than type 2, and type 1 is more stable on the cell surface and is less likely to be excised and released into the blood^10^. These results indicated that protein restriction did not affect the abundance ratios of type 1 and type 2.

It has been reported that an increase in hepatic *Vldlr* mRNA is also caused by ER stress induced by tunicamycin administration, and the increase in *Vldlr* mRNA is mediated by ATF4 activation^12^. Protein deficiency also activates ATF4 through decreased translational activity mediated by general control nonderepressible 2 (GCN2) and eukaryotic translation initiation factor 2 (eIF2)^26^. ATF4 activation in mice fed a low-protein diet has been reported from the results of liver DNA microarray analysis^22^. It has been reported that feeding low-protein diet to mice increases XBP-1 splicing, but has no effect on ATF6 levels^27^. These results indicate that not all ER stress pathways are activated by protein deficiency, and it is highly likely that the ATF4 activation observed in this study is caused by the GCN2-eIF2 pathway due to protein deficiency. Therefore, the amount of *Asns* mRNA in protein-restricted mice, which is known to be upregulated by ATF4 activation^26^, was measured in this study. The results demonstrated a liver-specific increase in *Asns* mRNA, indicating that ATF4 was strongly activated only in the liver. In WT mice, *Vldlr* mRNA and *Asns* mRNA levels were significantly correlated in the liver, but not in other organs (Spearman’s rank correlation coefficient (r) and significance probability (*P*) were as follows: r = 0.6737, *P* < 0.01 in liver, r = 0.4097, *P* = 0.0913 in heart, r = 0.2368, *P* > 0.1 in muscle, r = 0.4536, *P* = 0.0895 in eWAT). Therefore, protein restriction strongly induced liver-specific ATF4 activation, which might lead to an increase in VLDLR expression. Increased FGF21 by ATF4 has also been reported to promote VLDLR synthesis^14^; however, the current results showing that hepatic *Vldlr* mRNA is increased by protein restriction in *Fgf21* KO mice suggests otherwise (Fig. S2).

It has long been known that protein deficiency causes fatty liver, which is also observed in kwashiorkor, a protein deficiency in human children^23^. There are multiple factors that cause fatty liver, such as decreased VLDL secretion from the liver^28–30^ and increased hepatic lipid synthesis^31,32^. It has also been reported that hepatic insulin signaling is enhanced despite reduced insulin secretion, which may cause increased lipogenesis in protein-restricted rats^33,34^. The results of this study showed that hepatic lipid accumulation due to protein restriction was also observed in *Vldlr*-deficient mice, indicating that VLDLR was not a direct cause of fatty liver. The result that there was no decrease in plasma TG and cholesterol levels due to protein restriction also indicated that lipid uptake from blood was not the cause for the increased hepatic fat. It was also confirmed that the increase in hepatic fat due to protein restriction was not suppressed in *ApoE*-deficient mice (Fig. S3), which also supported the hypothesis that VLDL uptake into the liver was not a major factor in fatty liver during protein restriction. The secretion assay results using a lipoprotein lipase inhibitor, and the results obtained via the fatty acid uptake assay, have demonstrated that lipid secretion from the liver and fatty acid uptake into the liver are not altered by protein deficiency^35^. Therefore, fatty liver due to protein deficiency is likely to be caused by multiple factors, such as the influx of excess energy into the liver due to loss of muscle mass^22^ and increased lipid synthesis in the liver^31,32^. Considering that VLDLR contributes to fatty liver formation in ER stress and alcoholic fatty liver^12,13^, it is assumed that the increased VLDLR in protein-restricted mice may contribute to fatty liver formation. However, the contribution of VLDLR is possibly small compared to other factors described above in the case of protein restriction, especially in the early stages of deficiency, because the increase in *Vldlr* mRNA began to appear after the onset of increased liver fat in rats (Fig. S4); therefore, the effect of *Vldlr* deficiency may become visible in experiments with a longer time period. We are currently examining this possibility.

The liver is usually an organ with low VLDLR expression^10,11^. The results of western blot analysis revealed that the amount of VLDLR in the liver of protein-restricted mice was lower than that in the heart, muscle, and adipose tissue of the control mice (Fig. S1). Therefore, the amount of VLDL taken up by the liver of protein-restricted mice was not expected to be higher than that of other organs. In Fig. S3, type 1 and type 2 VLDLRs are detected in eWAT, which is different from the result in Fig. 3D. This is probably due to the use of different antibodies.

Since the role of VLDLR in liver fat during protein restriction was not apparent, its role in inflammation was also examined. Blood AST concentration was increased by protein restriction only in *Vldlr* KO mice, demonstrating that VLDLR might suppress liver damage in protein-deficient mice. However, it could not be concluded that increased VLDLR in protein deficiency affected inflammation because plasma ALT concentration or expression of inflammatory marker genes in the liver was not affected by *Vldlr* deficiency. In addition, many studies have reported that VLDLR promotes inflammation, and few suppress it^36^. Further studies are required to clarify the role of VLDLR in the liver under protein-restriction.

In conclusion, it was demonstrated that *Vldlr* mRNA and VLDLR protein levels were increased by dietary protein restriction in a liver-specific manner in mice. The liver-specific increase was probably due to the activation of ATF4. Although ER stress-induced fatty liver is caused by increased VLDLR^12^, the results with *vldlr* KO mice revealed that increased VLDLR is not a major factor in fatty liver formation during protein restriction. We have clarified the characteristic of fatty liver formation due to protein deficiency, which is caused by multiple factors other than increased VLDLR.

## Methods

### VLDLR knockout mice

Homozygous KO (*Vldlr*^−/−^) mice were purchased from the Jackson Laboratory (No.002529 strain B6;127S7-Vldlr < tm1Her > /J) and bred with C57BL/6 J WT (*Vldlr*^+/+^) mice (Japan SLC, Inc.) to obtain heterozygous KO (*Vldlr*^+/−^) mice. The *Vldlr*^+/−^ mice were then bred with *Vldlr*^+/−^ mice to obtain *Vldlr*^−/−^ (KO) and WT mice for subsequent experiments. Genotyping of animals was conducted after weaning, as described previously^22,37^ with the primers shown in Table 2. Animals were kept at 22–24 °C under a 12 h (06:00–18:00) light–dark cycle and allowed free access to tap water throughout the experiment. They were fed ad libitum a commercial pellet feed (Certified Diet MF; Oriental Yeast) before the feeding experiment.

### Animal experiments

Four-week-old male KO (n = 20) and WT (n = 20) mice were housed individually in stainless steel cages and fed a control diet with 20% casein as a protein source (20C, Table 1) for 3 days to acclimate to powdered feed. The KO and WT mice were then divided into two groups and fed 20P or a low-protein diet with 3% casein (3C, Table 1) for 11 days. Ten mice were assigned to each experimental group. On day 12, mice were anesthetized with 1 mL/kg intraperitoneal sodium pentobarbital (Somnopentyl; Kyoritsu Seiyaku), heparinized blood was collected by cardiac puncture, and plasma samples were obtained. Liver, heart, epididymal white adipose tissue (epi-WAT), and gastrocnemius muscle were excised, weighed, frozen in liquid nitrogen, and stored at -80 °C until analysis. Animal experiments were approved by the Meiji University Institutional Animal Care and Use Committee (Approval Number: IACUC 15-0007) and performed in accordance with the University guidelines. Additional details of mice and experimental procedures are included in the Supplementary information file. This study was carried out in compliance with the ARRIVE guidelines.

**Table 1:** Composition of the diets.

	20C	3C
	(g/100 g diet)	
Casein	20	3
L-Methionine	0.32	0.048
Corn oil	5	5
α-Cornstarch	43.45	54.96
Sucrose	21.73	27.49
Cellulose	5	5
AIN 93 vitamin mixture	1	1
AIN93G mineral mixture	3.5	3.5
Total	100	100

### RNA preparation and real-time PCR

Total RNA was extracted from tissues, and used for cDNA synthesis and real-time PCR using the TriPure Isolation Reagent (Roche Applied Science), PrimeScript RT reagent Kit with gDNA Eraser (Perfect Real Time), and THUNDERBIRD SYBR qPCR Mix (Toyobo), respectively, according to the manufacturers’ instructions^38^. β-actin was used as an internal control. Amplification of a single PCR product for each primer set was confirmed using melting curve analysis. Each result was normalized to the average value of the control group and expressed as relative mRNA levels. Primer sequences are shown in Table 2.

**Table 2:** Primer sequences and product sizes of the RT-PCR.

Gene name	5′-sequence-3′	Product size (bp)
**Realtime PCR**		
Actb (β-actin)		
Forward	AAGTGTGACGTTGACATCCGTAA	
Reverse	GCAATGCCTGGGTACATGGT	
Asns		
Forward	TTGCTGCCTCTCTGCTGAAG	
Reverse	GTCCTCCATGCCAATAGCAAA	
Gusb		
Forward	ACATGCCTGTCCCTTCTAGCTT	
Reverse	TTCATACCACACCCAGCCAAT	
Hprt1		
Forward	GCCGAGGATTTGGAAAAAGTG	
Reverse	TTCATGACATCTCGAGCAAGTCTT	
IL-1β		
Forward	AAGATGAAGGGCTGCTTCCA	
Reverse	ATGTGCTGCTGCGAGATTTG	
IL-6		
Forward	TTCTCTGGGAAATCGTGGAAA	
Reverse	TGCAAGTGCATCATCGTTGTT	
Tnf-α		
Forward	AGGGTCTGGGCCATAGAACT	
Reverse	CCACCACGCTCTTCTGTCTAC	
Vldlr		
Forward	TGACGCAGACTGTTCAGACC	
Reverse	GCCGTGGATACAGCTACCAT	
Type1-Vldlr		
Forward	TCAGAGCTGGCCACTCTAGTGA	
Reverse	CCTGACGGCTGGACGAGTT	
**RT-PCR**		
Vldlr exon16-spanning primers		
Forward	ATATCTCTGCCTGCCAGCACC	230 and 314
Reverse	TCCTCCACATCAAGTAGCCACC	
**Genotyping**		
Vldlr		
WT Forward	TGGTGATGAGAGGCTTGTATGTTGTC	
WT Reverse	TTGACCTCATCGCTGCCGTCCTTG	
Mutant Forward	CGGCGAGGATCTCGTCGTGACCCA	
Mutant Reverse	GCGATACCGTAAAGCACGAGGAAG	

### Protein extraction and western blotting

Protein extraction from tissues was performed, as described previously^39^, except for the use of 250 μL homogenizing buffer for 70 mg eWAT samples. Protein samples were frozen immediately after extraction in liquid nitrogen and stored at − 80 °C until use. Sodium dodecyl sulfate-polyacrylamide gel electrophoresis and western blotting were performed, as described previously^38^. Anti-VLDLR antibody (Novus Biologicals, NBP1-78162, 1:600 dilution for Fig. 3BD; R&D systems, AF2258, 1:2000 dilution for Figs. 3A,C and S3), β-actin antibody (Abcam, ab6276, 1:500 dilution), and anti-α-tubulin antibody (Abcam, ab4074, 1:3000 dilution) were used as primary antibodies. The secondary antibodies, goat anti-mouse IgG-horseradish peroxidase (HRP, Abcam, ab205719, 1:50,000 dilution), donkey anti-goat IgG-HRP (Santa Cruz, sc2020, 1:10,000 dilution) and goat anti-rabbit IgG-HRP (Abcam, ab6721, 1:10,000 dilution) were used for detection of VLDLR and β-actin, and α-tubulin, respectively. Luminescence was detected using Immobilon Western Chemiluminescent HRP Substrate (Merck) and an image analyzer (ImageQuant LAS 4000 mini, GE Healthcare Life Sciences, Illinois, USA). The results were digitized using Image J (National Institute of Health) and all values were normalized to the average value of the WT20C group and expressed as relative protein levels. Blot densitometry band quantification was performed using unmodified images. Quantitative values for samples from different membranes were standardized based on values from the same control sample applied to all gels. Figure 3 contains some cropped images, and Figs. S1 and S6 show full-size unmodified images.

### RT-PCR

The two types of *Vldlr* mRNA splicing variants, type 1 and type 2, generated by alternative splicing of exon 16, were analyzed by RT-PCR with Blend Taq (Toyobo) and primers sandwiching exon 16 according to the method of Sakai et al. (primer sequences are shown in Table 2^19^). Total RNA extraction from the liver and cDNA synthesis were performed as described above. It was confirmed that the amplification did not reach a plateau under the 30-cycle condition used for measurement. Figure 2E contains a cropped image, and Fig. S5 shows a full-size unmodified image.

### Plasma and liver lipid analyses

Total lipid was extracted from tissues using the method of Bligh and Dyer^40^ and reconstituted in isopropanol. Triacylglycerol, total cholesterol, and phospholipids in plasma, and total tissue lipid samples were measured using the Triglyceride E-Test Wako (Wako Pure Chemicals), Cholesterol E-Test Wako (Wako Pure Chemicals), and Phospholipid C-Test Wako (Wako Pure Chemicals), respectively.

### Markers of liver injury

Plasma aspartate aminotransferase (AST) and alanine aminotransferase (ALT) concentrations were determined using the contract measurement service provided by Oriental Yeast Co., Ltd.

### Statistical analysis

To analyze the differences between 20 and 3C, Student’s *t*-test or Welch’s *t*-test was performed for data with equal or unequal homogeneity of variance, respectively. To analyze the data on body weight, food intake and plasma TG levels, two-way analysis of variance was performed to evaluate the significant effects of *Vldlr* deficiency, low protein diet, and the interaction of VLDLR × diet on the values shown in Fig. 1A,B. A *P* value < 0.05 was considered significant. All statistical analyses were performed using Excel statistics software 2016 (Bell Curve).

## Supplementary Information

Supplementary Information.
