# Supplementary material for: Very low-density lipoprotein receptor increases in a liver-specific manner due to protein deficiency but does not affect fatty liver in mice
Source: Sci Rep. 2021 Apr 13;11:8003. doi: 10.1038/s41598-021-87568-2 (PMC8044231; doi:10.1038/s41598-021-87568-2)
Supplement: Supplementary file 1 — Supplementary Information. [file 41598_2021_87568_MOESM1_ESM.docx]

**Very low-density lipoprotein receptor increases in a liver-specific manner due to protein deficiency but does not affect fatty liver in mice**

Yui Oshio^1^, Yuta Hattori^1^, Hatsuho Kamata^1^, Yori Ozaki-Masuzawa^2^, Arisa Seki^1^, Yasutaka Tsuruta^1^, Asako Takenaka^1^*

1Department of Agricultural Chemistry, School of Agriculture, Meiji University, Kawasaki, Kanagawa 214-8571, Japan

2Department of Chemistry and Life Science, College of Bioresource Sciences, Nihon University, Kameino, Fujisawa, Kanagawa, Japan

* corresponding author

Asako Takenaka

Department of Agricultural Chemistry, School of Agriculture, Meiji University, Kawasaki, Kanagawa 214-8571, Japan

E-mail: [takenaka@meiji.ac.jp](mailto:takenaka@meiji.ac.jp)

**Supplementary Methods**

*Animal experiments*

Fig. S2: To evaluate the effect of Fgf21 deficiency and dietary protein restriction on *Vldlr* mRNA levels, 4–5 week-old C57BL/6J male mice (WT) and *Fgf21*-deficient male mice (KO) were fed a control diet with 20% protein (20C) or the low protein diet with 5% protein (5C) for 11 days, as previously reported ^25^.

Fig. S3: The effect of ApoE deficiency and dietary protein restriction on liver TG levels was determined in 4-week-old C57BL/6J male mice (WT) and *ApoE*-deficient male mice B6.SHL (*ApoE*-/-) (Japan SLC), fed a control diet with 20% protein (20C) or a low protein diet with 5% protein (5C) for 10 days. Five rats per each experimental group were used. Details of the animal experiment methods are the same as those described in manuscript.

Fig. S4: Changes in *Vldlr* mRNA content and liver TG over time during protein deficiency were assessed in 5-week-old Wistar male rats, fed a control diet with 20% protein (20C) or a low protein diet with 5% protein (5C) for 1, 2, 3, or 7 days. The food intake of the rats was paired with the 5C group. Five or six rats per each experimental group were used. Details of the animal experiment methods are the same as those described in manuscript.

**Supplementary Figures**

**Supplementary Figure S1.** The amount of very low-density lipoprotein receptor (VLDLR) in the liver, heart, muscle, and epididymal white adipose tissue (eWAT) of WT mice fed the control (20C) or protein-restricted (3C) diet.

Protein samples (48.75 μg) from liver, heart, muscle, and eWAT of WT mice, as analyzed by western blotting. A typical image of the blot is shown. The positions of the type 1 and type 2 bands are shown on the left of the image and that of the molecular weight marker is shown on the right.

**Supplementary Figure S2.** Effect of fibroblast growth factor 21 (*Fgf21*) deficiency and dietary protein restriction on liver very low-density lipoprotein receptor (*Vldlr*) mRNA.

The liver *Vldlr* mRNA content was measured in C57BL/6J male mice (WT) and *Fgf21*-deficient male mice (KO), 4–5 weeks old, fed a control diet with 20% protein (20C) or the low protein diet with 5% protein (5C) for 11 days. Details of animal experiments have previously been reported ^25^. Values are expressed as a relative value with the WT 20C group as 1, the mean ± SEM, n = 5 or 6 per group. **P* < 0.05 vs. 20C.

**Supplementary Figure S3.** Effect of *ApoE* deficiency and dietary protein restriction on liver triglycerides (TG).

C57BL/6J male mice (WT) and *ApoE*-deficient male mice B6.SHL (*ApoE*-/-) (Japan SLC), 4 weeks old, were fed a control diet with 20% protein (20C) or a low protein diet with 5% protein (5C) for 10 days, and the amount of liver TG was measured. Values are expressed as a relative value with the WT 20C group as 1 and the mean ± SEM, n = 5 per group. ***P* < 0.01, **P* < 0.05 vs. 20C.

**Supplementary Figure S4.** Changes in *Vldlr* mRNA content and liver triglycerides (TG) over time during protein deficiency

Wistar male rats, 5 weeks old, were fed a control diet with 20% protein (20C) or a low protein diet with 5% protein (5C) for 1, 2, 3, or 7 days. Food intakes of the rats were paired with the 5C group. Five or six rats per each experimental group were used. The amount of liver TG (A) and the relative amount of *Vldlr* mRNA (B) were measured. *Vldlr* mRNA levels are expressed as a relative value with the 20C group as 1. All values are expressed as mean ± SEM, n = 5 or 6 per group. ***P* < 0.01, **P* < 0.05 vs. 20C.

**Supplementary Figure S5.** Original image of the gel in Figure 2E.

**Supplementary Figure S6.** Original images of the blots in Figure 3A-D.

The images of tubulin in Figure 3B were only saved in a form that did not include the edges of the membranes, and only the cropped images are provided.
